# Does Implant Coating With Antibacterial-Loaded Hydrogel Reduce Bacterial Colonization and Biofilm Formation in Vitro?

**DOI:** 10.1007/s11999-014-3558-1

**Published:** 2014-03-13

**Authors:** Lorenzo Drago, Willemijn Boot, Kostantinos Dimas, Kostantinos Malizos, Gertrud M. Hänsch, Jos Stuyck, Debby Gawlitta, Carlo L. Romanò

**Affiliations:** 1 Dipartimento di Chirurgia Ricostruttiva e delle Infezioni Osteo-articolari, Istituto Ortopedico IRCCS Galeazzi, Via Riccardo Galeazzi, 4, Milano, 20161 Italy; 2 Department of Orthopaedics, University Medical Center Utrecht, Utrecht, The Netherlands; 3 University Hospital of Larissa, Larissa, Greece; 4 Institut für Immunologie, Universität Heidelberg, Heidelberg, Germany; 5 Department of Orthopaedic Surgery, Leuven University, Leuven, Belgium

## Abstract

**Background:**

Implant-related infections represent one of the most severe complications in orthopaedics. A fast-resorbable, antibacterial-loaded hydrogel may reduce or prevent bacterial colonization and biofilm formation of implanted biomaterials.

**Questions/purposes:**

We asked: (1) Is a fast-resorbable hydrogel able to deliver antibacterial compounds in vitro? (2) Can a hydrogel (alone or antibacterial-loaded) coating on implants reduce bacterial colonization? And (3) is intraoperative coating feasible and resistant to press-fit implant insertion?

**Methods:**

We tested the ability of Disposable Antibacterial Coating (DAC) hydrogel (Novagenit Srl, Mezzolombardo, Italy) to deliver antibacterial agents using spectrophotometry and a microbiologic assay. Antibacterial and antibiofilm activity were determined by broth microdilution and a crystal violet assay, respectively. Coating resistance to press-fit insertion was tested in rabbit tibias and human femurs.

**Results:**

Complete release of all tested antibacterial compounds was observed in less than 96 hours. Bactericidal and antibiofilm effect of DAC hydrogel in combination with various antibacterials was shown in vitro. Approximately 80% of the hydrogel coating was retrieved on the implant after press-fit insertion.

**Conclusions:**

Implant coating with an antibacterial-loaded hydrogel reduces bacterial colonization and biofilm formation in vitro.

**Clinical Relevance:**

A fast-resorbable, antibacterial-loaded hydrogel coating may help prevent implant-related infections in orthopaedics. However, further validation in animal models and properly controlled human studies is required.

## Introduction

Once a biofilm has been formed on an implant’s surface, it is difficult to treat the infection because the bacteria residing in the biofilm are protected from both phagocytosis and antibiotics. Evidence shows the role of biofilms as an impenetrable mechanical barrier against soluble agents [5, 34, 42] and this explains why, over the last decades, systemic antibiotics have shown their limits in treatment and prevention of biofilm-related infections [28, 29, 42].

To prevent bacterial colonization of implanted biomaterials, various antibacterial surface coatings have been proposed, but current technologies are far from large-scale application in orthopaedics due to various limitations, including questionable long-term effect on bacterial resistance and on bone ingrowth, regulatory issues, and costs [1, 4, 17, 23, 37, 39]. In this evolving panorama, a fast-resorbable antibacterial-loaded hydrogel coating may theoretically offer (1) efficacy toward early bacterial colonization, providing complete protection of the implant for the time needed to win the “race to the surface,” ie, in the first hours after surgery; (2) safety, as high local concentration and fast and complete release of the antibacterial may avoid induction of antibiotic resistance and possible risks of long-term effects on bone healing; (3) versatility, through intraoperative mixing with a choice of different antibacterial agents; (4) ease of handling; and (5) reduced costs for large-scale application.

In this preclinical multiinstitutional study, we investigated (1) the ability of a fast-resorbable hydrogel to deliver different antibiotic and antibiofilm compounds locally; (2) the ability of the hydrogel coating, alone and in combination with antibacterial agents, to reduce or prevent bacterial colonization and biofilm formation on biomaterials commonly used in orthopaedics in vitro; and (3) the feasibility of intraoperative coating of a standard joint prosthesis and the capability of the coating to resist press-fit intramedullary implant insertion.

## Materials and Methods

All reported experiments were performed using the Disposable Antibacterial Coating (DAC) (Novagenit Srl, Mezzolombardo, Italy) hydrogel, a patented, Conformité Européene (CE)-marked medical device, intended to be used as a disposable, fast-bioresorbable antibacterial coating for implants such as joint prostheses or osteosynthesis. DAC hydrogel is not cleared for use in the United States by the FDA.

**Fig. 1:**
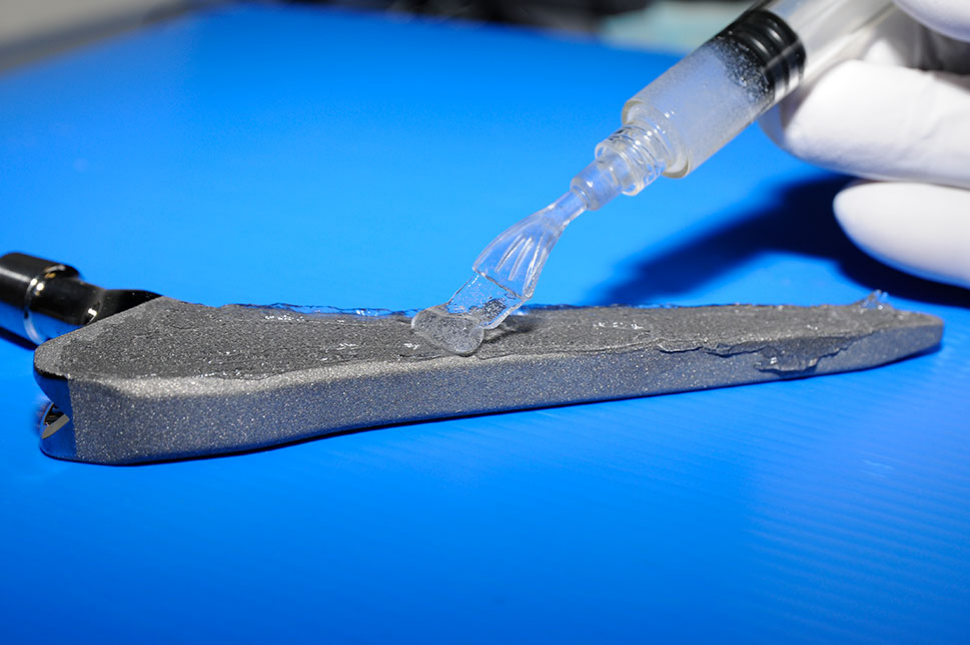
Spreading of the hydrogel on a titanium prosthesis through a suitable syringe spreader is shown.

DAC hydrogel, previously shown to meet the UNI EN ISO 11137-10993-1, 10993-3, 10993-5, 10993-6, 10993-9, 10993-10, 10993-11, 10993-13, ISO 13781:1997, ASTM F 1635-11 standards for safety (Novagenit Srl, data on file), is composed of covalently linked hyaluronan and poly-D,L-lactide; complete hydrolytic degradation of the hydrogel is supposed to take place in vivo [35]. The hydrogel was delivered in a fully functional kit, comprising two separately packaged units: (1) a syringe containing the DAC^®^ powder, ready for reconstitution with the active drug, and (2) a procedure pack containing three components for reconstitution and spreading of the resulting hydrogel (Fig. 1).

The present research was conducted under the multiinstitutional collaborative project “Implant Disposable Antibacterial Coating (IDAC): A Novel Approach to Implant-Related Infections in Orthopaedics and Trauma Surgery,” funded by the European Commission, within the Seventh Framework Programme on Research Technological Development and Demonstration under Grant 277988.

### Antibiotic Delivery Ability

Studies were performed to evaluate the ability of this hydrogel to release different antibiotics and antibiofilm compounds. Double-packaged syringes containing sterile DAC^®^ powder were provided by the manufacturer. The reconstituted hydrogel was studied with regard to its ability to deliver bactericidal levels of a selection of antibiotic and antibiofilm compounds (vancomycin, gentamicin, tobramycin, amikacin, N-acetylcysteine [NAC], and sodium salicylate). All of these compounds were purchased from Santa-Cruz Biotechnology Inc (Santa Cruz, CA, USA) in the form of powder, except for gentamicin, which was obtained in solution at a concentration of 50 mg/mL.

Photometric measurements were performed by means of a Cobas Integra^®^ 400 Plus analyzer (Roche Diagnostics Ltd, Rotkreuz, Switzerland) at the Laboratory of Clinical Pharmacology of the University Hospital of Larissa (Larissa, Greece). Each release experiment was performed twice.

Reconstitution of the gel was performed according to the manufacturer’s instructions. Briefly, syringes prefilled with 60 mg DAC^®^ powder were reconstituted with 1 mL of sterile water containing the antibacterial or antibiofilm substances to obtain a hydrogel with a DAC^®^ concentration of 6% (w/v).

The final concentration used for all agents mixed with 1 mL hydrogel was 20 mg/mL, except for tobramycin, which was used at a concentration of 10 mg/mL. Time points used in the in vitro release study were 2, 4, 6, 24, 48, and 96 hours. All release studies were performed in fetal calf serum (FCS) (Biowest SAS, Nuaillé, France) in Nunc^®^ six- and 48-well culture plates (Thermo Scientific, Milano, Italy). Vancomycin release was also studied in human serum (HS) (Life Technologies Corp, Grand Island, NY, USA) at two starting concentrations of 20 mg/mL and 2 mg/mL.

Hydrogel coating of different biomaterial surfaces (cobalt-chrome disks, polyethylene disks, titanium disks, and plastic culture well surfaces) was tested after loading with vancomycin, gentamicin, amikacin, tobramycin, NAC, and sodium salicylate, according to the same procedure. Briefly, a solution of the substance to be tested was prepared using water for injections, taking into account the indicated amount to be loaded in the hydrogel and assuming that the powder reconstitution was carried out directly with this solution. Then, 1000 μL solution containing the substance to be tested was taken with a syringe that was connected to a syringe containing DAC powder to allow the reconstitution according to manufacturer’s instructions. After complete hydration of the product, the disk simulating the surface of the orthopaedic implant under study was placed on an analytical balance to a weight of 200 mg (10% tolerance) of gel. This quantity of gel was then spread uniformly over the entire surface of each disk using the spreader supplied together with the other components. The disk with the gel was then immersed in 6 mL FCS or HS and the container closed to prevent evaporation and stored at 37° C without shaking. Then, 1 mL release medium was removed with a precision pipette and sterile tips at 2, 4, 6, 24, 48, and 96 hours. The 1-mL aspired aliquot was placed in a 2-mL plastic vial and frozen at −20° C for subsequent analysis. The release medium collected was immediately replaced by 1 mL fresh FCS or HS, so that the volume was kept at 6 mL until the end of the study.

Release data for the single experimental time points were calculated as follows: analytical raw data (expressed as μg/mL) normalized to the instrumental standard curve were multiplied with the total buffer volume (mL) used for incubation to determine substance quantity (μg). The incubation volume was kept constant throughout the experiment by integrating at the moment of sampling the volume with fresh buffer. This procedure unavoidably leads to dilution. As a consequence, the overall quantity (in μg) for a given time point was determined by adding the amount of substance taken away in sampling the previous time points. Finally, the substance release was expressed as the percentage of the total quantity initially loaded (ie, concentration of the substance inside the hydrogel [μg substance/μg hydrogel] × quantity of hydrogel loaded on the disc [μg]).

In a separate experimental procedure, vancomycin concentration was also tested on sand-blasted titanium and chrome-cobalt disks (AdlerOrtho Srl, Milano, Italy) by means of a microbiologic assay, using a methicillin-resistant *Staphylococcus aureus* strain (MRBP-2) from the collection of the IRCCS Galeazzi Institute (Milan, Italy). The biologic measure of the antibiotic concentration was performed on the basis of its inhibitory effect (measurement of the inhibition zone diameter). To this aim, the inhibitory effect of the sample was compared with the inhibitory effect of graded doses of a standard. By using a calibration curve of different concentrations of antibiotic and calculating the regression equation, antibiotic concentration in elution fluids can be calculated. The following algorithm was used to calculate the antibiotic concentration:

$$ { \log }\left[ {{\text{antibiotic\; concentration }}\upmu {\text{g}}/{\text{mL}}} \right] \, = {\text{ intercept}} - {\text{slope}}\left[ {{\text{inhibition\; zone\; diameter }}\left( {\text{mm}} \right)} \right] $$

### Antibacterial Activity

The antibacterials tests were aimed at assessing the effects of the hydrogel coating, either pure or loaded with antibacterials, on bacterial growth and biofilm formation on common orthopaedic biomaterials.

#### Minimum Inhibitory Concentration

For the purpose of this study, minimum inhibitory concentration (MIC) was defined as the lowest concentration of antibacterial substance in the presence of which the tested microorganism was not able to grow. MIC values were determined by the broth microdilution method.

To evaluate any effect of the gel on the MIC values, the test was conducted for each strain and substance on the gel alone, on the antibacterial/antibiofilm substance alone, and on the gel supplemented with the antibacterial/antibiofilm substances.

Gentamicin, vancomycin, and NAC were mixed into the gel to assess MIC tests and antibiofilm activities. These compounds were selected because gentamicin and vancomycin are among the most used antibiotics for local administration and also have different steric and chemical properties, so they may behave differently when mixed with the hydrogel. NAC was chosen as this is one of the few antibiofilm agents that is safe for parenteral use in humans and also has an antibacterial effect [17, 37], although it is not cleared for local administration in orthopaedics. Starting concentration, further diluted to test MIC, was 256 μg/mL for gentamicin and vancomycin and 100 mg/mL for NAC. Concentrations of the tested antibacterial were the same when it was tested alone or in combination with the hydrogel.

Clinical strains used in this study were selected from the Microbiology Laboratory collection (stored at −80° C) from patients of the Center for Reconstructive Surgery of Osteoarticular Infections of the IRCCS Galeazzi Institute. These strains were selected for their properties of resistance to antibacterial agents and for their high ability to produce biofilm on prosthetic materials in vitro. In particular, we used one clinical strain of methicillin-resistant *S aureus*, one of methicillin-resistant *Staphylococcus epidermidis*, one of *Escherichia coli*, one of vancomycin-resistant *Enterococcus faecalis*, one of *Acinetobacter baumannii,* and one of *Pseudomonas aeruginosa*.

All bacterial strains were grown overnight in tryptic soy broth (TSB) (Biomerieux, Marcy l’Etoile, France) at 37° C under aerobic conditions, unless specified otherwise.

Reconstitution of the gel was performed according to manufacturer’s instructions as described above.

For each strain, a bacterial suspension with a density equal to 0.5 McFarland (1 × 10^8^ colony-forming units [CFU]/mL) was prepared and properly diluted to obtain a concentration of 1 × 10^4^ CFU/mL; then 10 μL was inoculated in a 96-well microplate containing 100 μL TSB and a serial dilution of the tested substance. The last column of the plate was used as a positive control of growth. After incubation at 37° C for 24 hours, the MIC values were read, which corresponded to the last concentration in which there was visible bacterial growth by formation on the bottom. Assays were performed in duplicate for each strain, and if the MIC of two tests differed for more than one well, the assay was repeated.

#### Antibiofilm Activity

The same methicillin-resistant *S aureus* and *S epidermidis* strains as indicated above were used to evaluate the antibiofilm activity on mature biofilm of the gel reconstituted with vancomycin, gentamicin, and NAC. The hydrogel supplemented with vancomycin (20 mg/mL), gentamicin (20 mg/mL), or NAC (100 mg/mL) was compared to each antibacterial alone.

Sand-blasted titanium, cobalt-chrome, and polyethylene disks were used as substrate for biofilm formation. Briefly, the disks were placed into six-well flat-bottomed sterile polystyrene microplates (Jet Biofil^®^; Guangzhou Jet Bio-Filtration Products Co, Ltd, Guangzhou, China) containing 5 mL TSB and 200 μL of the bacterial suspension. The microplates were incubated at 37° C aerobically. After 24 hours, the exhausted growth medium eventually containing the nonadherent bacteria was removed and replaced by 5 mL fresh medium. The plates were incubated for a further 48 hours to obtain the mature biofilm. Before the treatments, the remaining nonadhering bacteria, if any, were removed by washing three times with sterile saline solution. For each strain, several disks were prepared for monitoring the hydrogel activity at different time points: 2, 4, 6, 24, and 48 hours after the biofilm-gel contact. Two hundred milligrams (10% tolerance) of gel was spread over the entire surface of each disk with mature biofilm and each disk was incubated under proper conditions with 5 mL fresh TSB. At each time point, disks were recovered and, after several washes, allowed to air dry. Subsequently, to evaluate the efficacy of the hydrogel supplemented with antibacterial or antibiofilm substances, the whole biomass present on each disk was determined after different incubation times by the method described by Christensen et al. [12]. Briefly, air-dried disks were immersed in a 5% crystal violet solution for 15 minutes and, after several washings, were air dried again. The estimation of biofilm biomass was performed by elution of the biofilm bound to crystal violet with 3 mL ethanol (96%) followed by the determination of the absorbance of 100 μL of eluted dye solution at 595 nm using a microplate photometer (Multiskan FC™; Thermo Scientific). Measurements were carried out in triplicate. Percentage of biofilm reduction was calculated according to the following formula:

$$ \left[ {\left( {{\text{absorbance growth control}} - {\text{absorbance samples}}} \right)/{\text{absorbance growth control}}} \right]\times{\text{1}}00. $$

Two more *S aureus* and *S epidermidis* strains were tested at the University of Heidelberg to assess biofilm formation and bacterial growth inhibition on sand-blasted titanium at time intervals of 48 hours and 5 to 7 days, comparing the hydrogel alone to the gel supplemented with vancomycin (Calbiochem^®^; Merck KGaA, Darmstadt, Germany), gentamicin (Refobacin^®^; Merck KGaA), meropenem (Hospira; Hospira, Munich, Germany), rifampicin (Eremfat^®^; Fatol Arzneimittel GmbH, Schiffweiler, Germany), ciprofloxacin (Ciprobay^®^; Bayer HealthCare AG, Leverkusen, Germany), daptomycin (Cubin^®^; Cubist Pharmaceuticals, GmbH, Nuremberg, Germany), diclofenac sodium (Sigma-Aldrich, Munich, Germany), and NAC (Sigma-Aldrich) in concentrations ranging from 0.2 mg to 100 mg/mL.

### Surgical Use Feasibility

Experiments were performed to test the resistance of the hydrogel coating on an implant surface against removal after press-fit insertion in bone. These “drag tests” were performed in both an ex vivo animal model and human femurs.

#### Rabbit Tibias

Sixty milligrams of DAC powder was mixed with 1 mL water containing 1% methylene blue (Merck). The gel was then applied to a sand-blasted titanium rod (AdlerOrtho) (4.0-mm diameter, 25-mm length, mean 5.6-μm surface roughness) until the implant was completely covered. A hole with a diameter of 4.1 mm was drilled in the tibial medullary canals in the tibial plateaus of six rabbit previously explanted tibias (New Zealand White rabbits) (the tibias were kindly provided by the Central Laboratory Animal Research Facility of the University Medical Center Utrecht, Utrecht, The Netherlands). Each rod covered with gel was inserted into one of the tibias. The tibias were sawed in half with an electrical saw. Photographs were taken of the implants and of both sides of the tibias. To calculate the amount of hydrogel that remained on the implants, the implant alone and the implant covered with hydrogel before and after implantation were weighed, as well as the amount of gel that was squeezed out of the canal after inserting the titanium rod.

#### Human Femurs

In 10 human femurs, the femoral head was removed with an oscillating saw and the femoral shaft reamed with dedicated instruments until the proper size for press-fit implant of a straight, sand-blasted (mean 6-μm surface roughness) titanium standard femoral stem (Recta; AdlerOrtho Srl) was achieved. Before implantation, 1% 300 mg DAC powder, reconstituted with 5 mL water for injections, containing 1% methylene blue (Merck) for further identification and also, in six femurs, vancomycin at a concentration of 2% w/v were applied on the prosthesis with a suitable spreader until the implant surface was completely covered. With 5 mL gel, it was possible to coat up to two medium-sized prosthetic stems. The spreading time ranged from 3 to 5 minutes. The implant covered with gel was then inserted into the femoral canal. The blue substance that squeezed out after implantation was collected and weighed. After press-fit insertion of the prosthesis, the femur was sawed in half longitudinally on the medial and lateral aspects using an oscillating saw, allowing opening of the shaft for inspection of the inner surface in contact with the prosthesis. Photographs were then taken of the implant and the femur on both sides and surface coverage by the hydrogel stained with methylene blue was visually analyzed. In the six femurs with vancomycin, the weight of the prosthesis before the coating and after explantation was compared to assess the amount of hydrogel on the prosthesis after insertion in the femoral canal.

### Statistical Analysis

Statistical analysis was conducted with a two-way ANOVA followed by Bonferroni’s correction using statistical software from VassarStats (Poughkeepsie, NY, USA). Significance level was set at p values of less than 0.05.

## Results

### Antibiotic Delivery Ability

**Fig. 2A–F:**
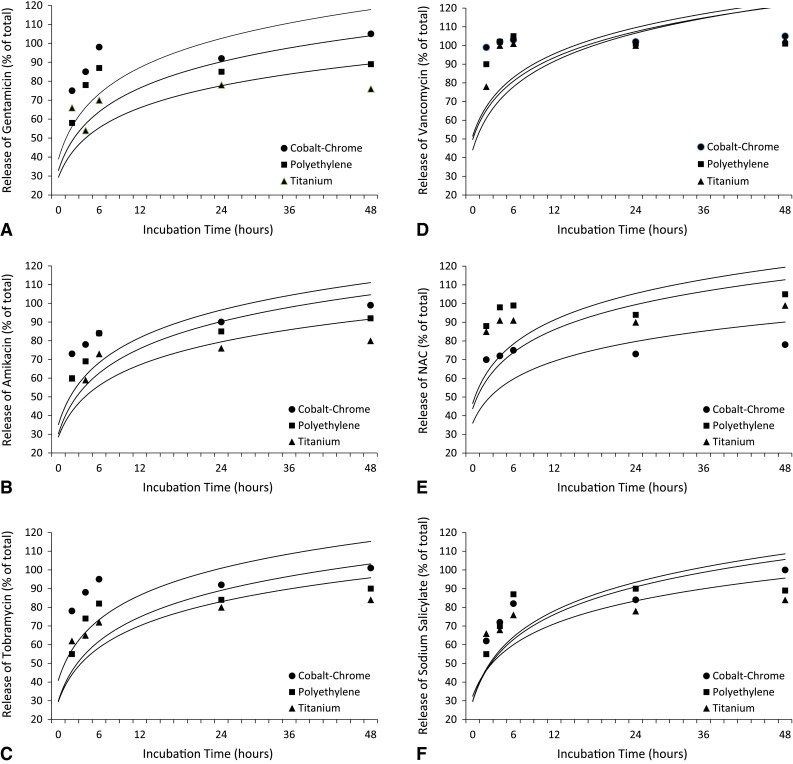
Graphs show the release kinetics of (**A**) gentamicin, (**B**) amikacin, (**C**) tobramycin, (**D**) vancomycin, (**E**) NAC, and (**F**) sodium salicylate from DAC hydrogel on different substrates (cobalt-chrome, polyethylene, titanium). Peak concentration was observed after 2 hours, regardless of the loaded compound and initial concentration.

Peak release was observed at 2 hours after submerging the coated disks into the serum, regardless of the compound, the surface, or the temperature at which incubation took place. The release patterns of gentamicin, amikacin, tobramycin, vancomycin, NAC, and sodium salicylate are reported on different substrates (Fig. 2). At 48 to 72 hours, the release of all tested compounds was almost complete or complete. The concentration at 96 hours was directly proportional to the initial concentration used to prepare the hydrogel. Thus, when 20 mg/mL was used as a starting concentration, the lowest level reached at 96 hours ranged from 200 to 300 μg/mL, while when an initial concentration of 2 mg/mL was used, the concentration at 96 hours was approximately 20 μg/mL.

Microbiologic assay showed, for vancomycin tested at an initial concentration of 20 mg/mL or 50 mg/mL, peak concentrations were approximately 1800 μg/mL and 3500 μg/mL, respectively, at 2 hours, with a gradual decline to 600 to 1000 μg/mL (polyethylene disks) or 350 to 540 μg/mL (cobalt-chrome disks) at 96 hours, respectively. Using the same approach and HS instead of FCS, peak concentration showed a slight delay (4 hours instead of 2 hours), while at 96 hours, final concentrations overlapped those measured in FCS.

In summary, both photometric measurement and microbiologic assays showed that all tested compounds were completely or nearly completely released from the hydrogel within 96 hours, with a peak release varying between 2 and 4 hours, depending on the medium, with concentrations measured at each time interval directly proportional to the starting ones.

### Antibacterial Activity

#### Minimum Inhibitory Concentration

The hydrogel alone did not show a measurable antibacterial activity, while the MICs for gentamicin, vancomycin, and NAC were unchanged or reduced up to four times when these compounds were tested in combination with the hydrogel (Table 1).

**Table 1:** Minimum inhibitory concentration of DAC hydrogel coating on a titanium surface alone or in combination with various antibacterial agents

Microrganism	Minimum inhibitory concentration (μg/mL)		Minimum inhibitory concentration (μg/mL)		Minimum inhibitory concentration (mg/mL)	
	Vancomycin	Hydrogel + vancomycin	Gentamicin	Hydrogel + gentamicin	N-Acetylcysteine	Hydrogel + N-acetylcysteine
*Staphylococcus epidermidis*	4*	1*	2*	0.5*	12.5	6.125
*Staphylococcus aureus*	0.5	0.5	2	1	25*	6.125*
*Enterococcus* *faecalis*	2*	0.5*	> 128	64	25*	6.125*
*Escherichia coli*	> 128	> 128	8	4	25*	6.125*
*Acinetobacter baumannii*	> 128	> 128	> 128	> 128	12.5	6.125
*Pseudomonas aeruginosa*	> 128	> 128	> 128	> 128	25*	6.125*

* A reduction of minimum inhibitory concentration of at least four times was observed using hydrogel + antibiotic in comparison to the antibiotic alone.

#### Antibiofilm Activity

**Fig. 3A–F:**
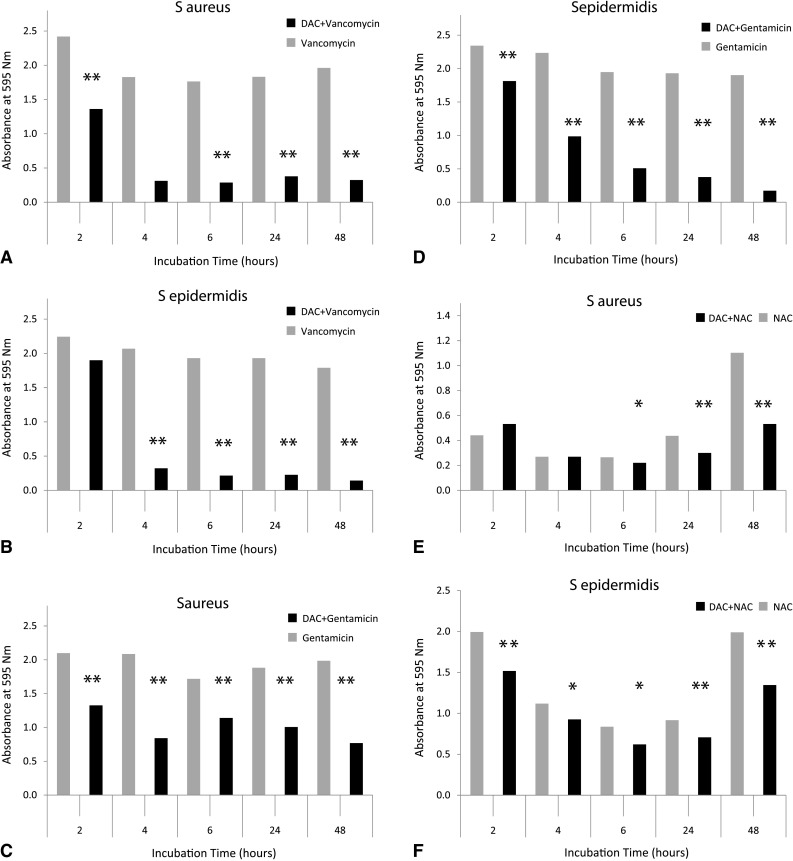
Graphs show antibiofilm activity on titanium disks of (**A**,** B**) vancomycin alone and hydrogel + vancomycin, (**C**,** D**) gentamicin alone and hydrogel + gentamicin, and (**E**, **F**) NAC alone and hydrogel + NAC in (**A**, **C**, **E**) *S aureus* and (**B**, **D**, **F**) *S epidermis*. Hydrogel supplemented with the different substances shows a greater antibiofilm activity when compared with the gel alone or with the substances alone (**p < 0.001; *p < 0.05).

At different time points, the hydrogel supplemented with vancomycin, gentamicin, or NAC reduced the amount of mature biofilm to a larger extent that that measured for any antibacterial alone on sand-blasted titanium disks (Fig. 3). The difference was evident from the very first hours of incubation and was maintained until the latest observation point of 48 hours. Similar results were obtained on polyethylene disks and chrome-cobalt substrates at 2 and 48 hours (Table 2).

**Table 2:** Antibiofilm effect of hydrogel supplemented with vancomycin, gentamicin, or N-acetylcysteine, compared with each antibacterial alone, tested on mature biofilm on polyethylene or cobalt-chrome disks

Hours of incubation	Absorbance at 595 Nm*											
	Polyethylene						Cobalt-chrome					
	*Staphylococcus aureus*			*Staphylococcus epidermidis*			*Staphylococcus aureus*			*Staphylococcus epidermidis*		
	Antiobiotic	Hydrogel + antibiotic	p value	Antibiotic	Hydrogel + antibiotic	p value	Antibiotic	Hydrogel + antibiotic	p value	Antibiotic	Hydrogel + antibiotic	p value
Vancomycin												
2	2.39 ± 0.10	1.31 ± 0.01	< 0.001	2.23 ± 0.02	1.23 ± 0.00	< 0.001	2.35 ± 0.11	1.28 ± 0.01	< 0.001	2.20 ± 0.02	1.81 ± 0.01	0.09
48	1.90 ± 0.03	0.30 ± 0.00	< 0.001	1.75 ± 0.02	0.14 ± 0.00	< 0.001	1.93 ± 0.02	0.29 ± 0.00	< 0.001	1.72 ± 0.02	0.13 ± 0.00	< 0.001
Gentamicin												
2	2.08 ± 0.04	1.30 ± 0.00	< 0.001	2.29 ± 0.00	1.82 ± 0.01	< 0.001	2.00 ± 0.05	1.28 ± 0.00	< 0.001	2.28 ± 0.00	1.66 ± 0.01	< 0.001
48	1.96 ± 0.02	0.76 ± 0.00	< 0.001	1.91 ± 0.01	0.16 ± 0.00	< 0.001	1.92 ± 0.02	0.75 ± 0.01	< 0.001	1.88 ± 0.01	0.17 ± 0.01	< 0.001
N-Acetylcysteine												
2	1.11 ± 0.01	0.92 ± 0.05	0.006	2.10 ± 0.01	1.49 ± 0.00	< 0.001	0.43 ± 0.04	0.45 ± 0.01	0.10	1.99 ± 0.00	1.48 ± 0.00	< 0.001
48	0.91 ± 0.00	0.70 ± 0.00	< 0.001	1.97 ± 0.02	1.31 ± 0.01	< 0.001	1.05 ± 0.00	0.51 ± 0.01	< 0.001	1.95 ± 0.02	1.33 ± 0.01	< 0.001

* Values are expressed as mean ± SD.

The hydrogel supplemented with various antibacterials showed a remarkable inhibition of biofilm formation and of planktonic bacterial growth of the tested strains, compared to the hydrogel alone (Table 3). Interestingly, pure antibiofilm agents, such as diclofenac sodium, only prevented biofilm formation but did not show any effect on planktonic bacteria growth. At the concentrations tested, NAC showed a progressively more effective bacterial growth inhibition and antibiofilm effect.

**Table 3:** *Staphylococcus aureus* biofilm and planktonic bacterial growth inhibition of DAC hydrogel loaded with various compounds, compared to hydrogel alone*

Antibiotic	Concentration (mg/mL)	Inhibition of biofilm growth compared to growth on hydrogel-covered titanium discs (%)		Inhibition of growth of planktonic bacteria (%)	
		Measured after		Measured after	
		48 hours	5–7 days	48 hours	5–7 days
Gentamicin	40	100	None	100	None
	10	25	None	None	None
Rifampicin	50	100	100	None	50
	10	100	100	None	40
Vancomycin	50	100	100	100	100
	10	100	100	100	100
Cibrofloxacin	1	100	100	100	100
Daptomycin	50	100	100	100	100
	10	100	100	100	100
Meropenem	50	100	100	100	100
	10	100	100	80	100
N-Acetylcysteine	20	100	100	100	100
	2	60	50	100	100
	1	60	50	100	100
	0.2	None	None	None	None
	20	100	100	20	None
	4	100	100	None	None
Diclophenac	20	100	100	20	None
	4	100	100	None	None

* All experiments were carried out on titanium discs; data are the mean of triplicates.

### Surgical Use Feasibility

#### Rabbit Tibias

After implantation of the gel-covered titanium rod, the mean ± SD amount of hydrogel adhering to the rod was 0.08 ± 0.01 g (range, 0.07–0.09 g) (58%) and the mean amount extruded during implantation was 0.06 ± 0.01 g (42%). On average, 0.24 mg/mm^2^ covered the surface of the rod after implantation.

After cutting the tibia in half, the implant showed methylene blue staining by the gel on the entire rod surface. The most intense blue staining however could be seen on both halves of the tibia. Interestingly, the staining on the inside of the tibia was most intense at the bottom and the upper part of the implant site. The middle part was either less intensely stained or not at all.

#### Human Femurs

**Fig. 4A–E:**
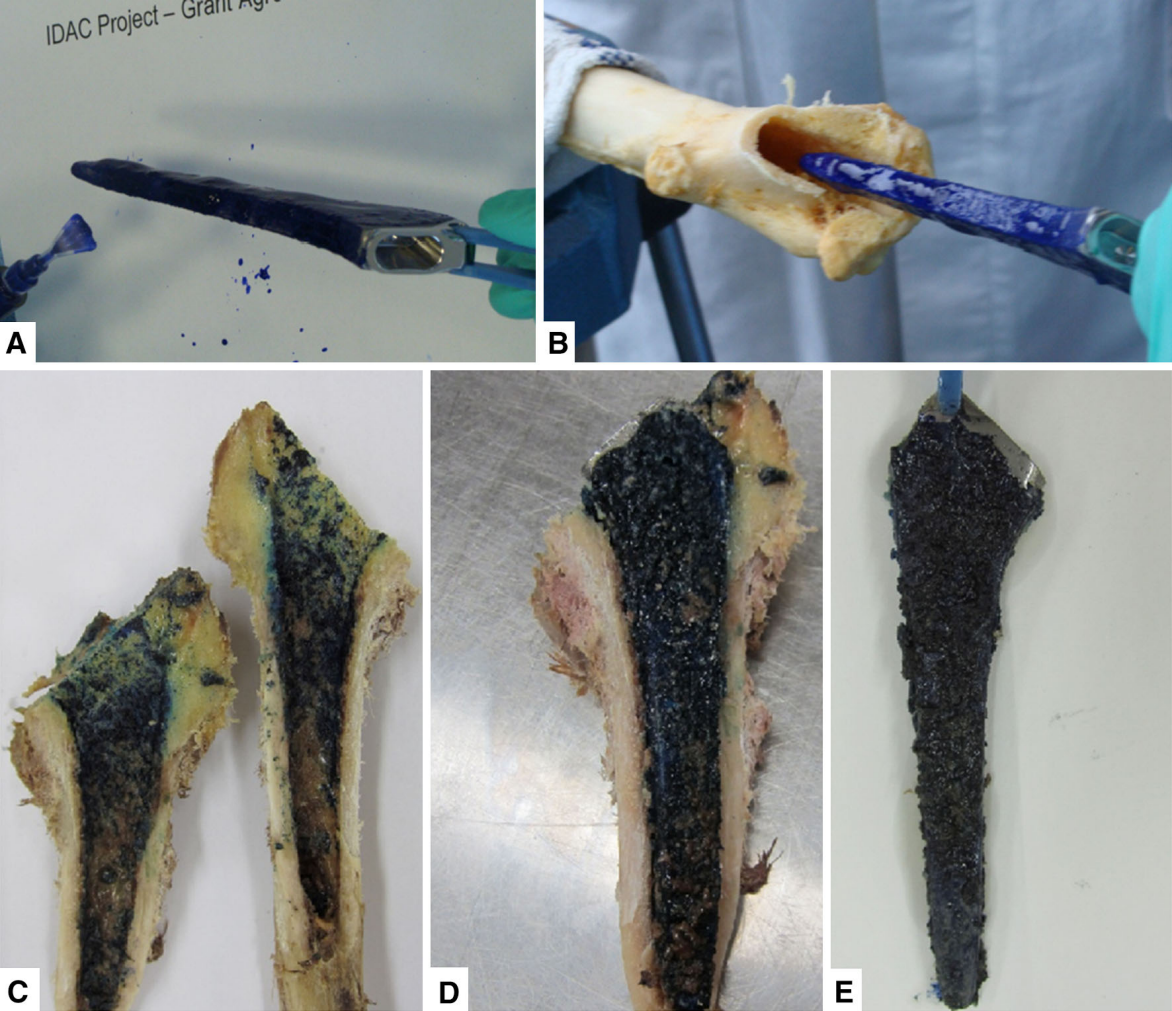
An implant (Size 6) was (**A**) covered with hydrogel and (**B**) implanted in a human femur. (**C**) Afterwards, the femur was cut into two halves. Note the blue staining heterogeneously distributed on the inner surface. (**D**, **E**) Both sides of the implant are completely covered with blue gel and some bone marrow (brown).

After implantation of the coated prosthesis and opening of the femurs, the implant appeared still completely covered with blue hydrogel. Furthermore, the inside of the femur was heterogeneously covered with blue hydrogel over the complete length of the implant (Fig. 4). Some of the bone marrow adhered to the corresponding place of the implant, which caused some absence of blue staining on the inside of the femur. Observing the residual traces of the dye on the inner side of the bone shafts, it was noted that most of the tissue staining appeared in areas of greater contact, ie, in the apical zones, corresponding to areas just below the greater trochanter. In deeper distal areas, it was noticed that different portions of the spongy bone had remained adherent to the stem of the prosthesis, confirming the remarkable adhesion of the hydrogel and the complete absence of visible dragging. On visual inspection, no difference in staining intensity at the surface of the implant was noted, comparing different experiments or when the hydrogel was used alone or mixed with 2% vancomycin.

Due to the mixing of the hydrogel with bone marrow, the substance that squeezed out during implantation together with the amount of gel covering the implant after implantation weighed more than the amount of gel applied before implantation. A mean of 78% ± 16% (range, 71%–85%) of the hydrogel initially applied to the prosthesis was retrieved on the explanted implants.

## Discussion

Implant-related infections represent one of the most severe complications in orthopaedics, with a reported incidence ranging from less than 1% to 3% after joint arthroplasty [9, 13, 30, 36] to 2% to 5% after spine surgery [14, 40] and even higher after severe trauma fixation [8, 31, 32]. To overcome this problem, various strategies to provide implants with an antibacterial coating have been proposed [19, 23]. A first approach includes porous materials, loaded with antibiotics; among these, antibiotic-loaded polymethylmethacrylate (PMMA) is probably the oldest and the best known. However, PMMA is a resin not originally designed to act as a local drug delivery carrier, is not suitable to coat osteosynthesis or cementless implants, is nonbiodegradable, and is prone to microbial adhesion and biofilm formation. Also, it may only be loaded with a restricted range of antibiotics and the long-lasting release may induce antibiotic resistance [27, 33, 38]. Other solutions, such as cancellous bone [10], collagen sponges [24, 24], or newer biodegradable elution systems [6, 26], share the goal of keeping the implant surface sterile and are biodegradable. Interestingly, most of these controlled-release systems are powerful therapeutic tools, showing high local antibiotic concentrations over a short term. For example, in an allograft system, independent of initial concentrations and time of impregnation, approximately 75% of the adsorbed vancomycin and approximately 99% of netilmicin elute within only 120 hours [41]. However, potential drawbacks of these elution systems when used as implant coatings include their limited antibacterial spectrum, possible exposure of the surviving bacteria to subinhibitory concentrations of antibiotics, unproven efficacy against biofilm-embedded bacteria or at preventing biofilm formation [15], possible local tissue toxicity and interference with implant osteointegration, and high costs [1, 4, 18, 25]. Another technologic approach, aimed at changing the physical/chemical composition of the implant surface permanently, such as silver-impregnated surfaces [20] or antibiotics or antimicrobial peptides covalently attached to an implant surface [2, 3, 39], raises still more concerns regarding long-term tissue toxicity, osteointegration, and bacterial-resistance induction, posing regulatory dilemmas that appear difficult to solve, as the implant becomes more and more similar to an active drug inserted into the body [11, 16]. Moreover, given the fact that the implant coating should be applied during manufacture, any new coating (eg, vancomycin instead of gentamicin) would require new investigations and approval by local regulatory bodies, with an exponential increase in time and costs.

In an effort to overcome at least some of the limits of the current approaches and instead of looking at long-term release systems, we investigated whether a fast-resorbable hydrogel was able to be loaded and to deliver antibacterial compounds locally, thus providing local antibacterial and antibiofilm protection in vitro and, at the same time, being capable of resisting declothing when used as a press-fit implant coating.

Our in vitro data show that all of the tested compounds were delivered from the hydrogel within 96 hours, with a peak in the very first hours. The quick time to a complete release of the antibacterial reduces to a minimum the risk of induced antibiotic resistance. It also represents a change of paradigm, where most researchers look for prolonged or permanent antibacterial coatings [23]; contrary to this common vision, our in vitro results support the concept that the race to the surface is won in the very first hours after implant insertion in the body [15] and that is the time when an antibacterial coating needs to exert its function, just as systemic prophylaxis has been shown to be necessary only in the short term perioperatively.

In line with this premise, a second goal of this study was to assess the ability of the antibacterial-loaded hydrogel to reduce or prevent bacterial colonization and biofilm formation on a coated implant in vitro. This is, to our knowledge, the first demonstration that a fast-resorbable biodegradable hydrogel was able to reduce or prevent biofilm formation in combination with commonly available antibiotics and antibiofilm agents. Concerning this latter achievement, a number of antibiofilm compounds are currently under study, but only NAC and some antiinflammatory drugs have shown antibiofilm activity and are cleared for human use, even if with different indications [37].

Furthermore, we addressed the ability of the hydrogel coating to resist press-fit insertion. Coatings of orthopaedics implants may in fact be detached, a problem both with controlled release and tethered systems. For all systems, the fragility is associated with the significant forces that are often applied to orthopaedic hardware during insertion [23]. In our experiments, we showed for the first time that a hydrogel coating may resist press-fit insertion in an animal model with a cylindrical nail and in a human femur model using a common press-fit femoral stem.

Our study had several major limitations. First, concerning the release studies, in our experimental condition, a rate of fluid exchange of approximately 1/6 of the initial volume at any fixed interval time was simulated; however, this is not necessarily what is be found in vivo, where these values are not necessarily the same and can vary greatly from one patient to another. Our results suggest that the tested hydrogel does not behave as a classical sustained-drug release system, as it was rapidly eluted in the serum, quickly releasing its content, ie, within the first 2 to 4 hours. The release profile was not altered, regardless of the compound tested, the medium, the surface that the hydrogel was applied to, or the incubation temperature. Further decrease of concentration of loaded agents appears to be dependent on starting values and on the rate of fluid exchange. It should be noted though that, in all the experimental conditions tested, despite the rapid release, the concentration remained much higher than the MIC (from 100 to 10 times higher) when 20 mg/mL was used. In the case of vancomycin, even when 2 mg/mL was tested as the starting concentration, the lowest levels obtained after 96 hours of incubation were close to the recommended trough levels for vancomycin (15–20 μg/mL; MIC for vancomycin, ≤ 2–4 μg/mL). Obviously, the final concentration is directly related to the starting concentration, which thus seems to be critical for clinical applications.

Concerning the antibacterial activity, a lower MIC for the antibiotic-loaded hydrogel compared to each substance tested alone (gentamicin, vancomycin, or NAC) was observed. However, this phenomenon has only been tested on a limited number of microorganisms and at a single antibacterial concentration. As previously published observations are lacking, further studies are needed to confirm these data and to evaluate its clinical relevance. Moreover, the possible mechanism underlying this finding, which may theoretically include increased cell permeability or longer stability and action of the drug delivered, has not been determined.

Hydrogel loaded with gentamicin, vancomycin, or NAC exhibited synergistic antibiofilm activity when compared with the activity of each agent alone against *S aureus* and *S epidermidis.* This finding is rather unique, since, although antifouling ability of hyaluronic acid has been previously reported, a possible synergistic effect of a hydrogel carrier and an antibacterial substance with regard to biofilm formation has not been described [7, 21]. Once again, the mechanisms underlying this finding have not been investigated and may be due to chemical and physical reasons. For example, in the case of NAC, the hydrogel may prevent oxidation, maintaining its antibiofilm activity for longer periods of time. However, we showed a synergistic antibiofilm effect of the hydrogel only in vitro, on a limited number of microorganisms and compounds, and at only one concentration. Further studies are needed to confirm these data and to evaluate their clinical relevance.

Utility in surgical situations is demonstrated by retention of hydrogel on coated implants during the implantation process. However, even if press-fit insertion in the diaphyseal canal of a sand-blasted titanium implant was not associated with declothing, both with or without vancomycin in the hydrogel coating, the ability of the hydrogel to coat other commonly used implants, such as acetabular cups, knee, or shoulder prostheses, plates, or nails, was not investigated and the effect of different surface finishing or composition is also open to further research.

In conclusion, our study provides evidence for the first time that a resorbable hydrogel, composed of covalently linked hyaluronan and poly-D,L-lactide, is able to quickly deliver local antibacterial compounds, thus inhibiting biofilm formation on different substrates and planktonic bacterial growth in vitro. Intraoperative coating of implants with the tested hydrogel appears safe and feasible, while resistance of the coating to scraping during press-fit implant insertion has been demonstrated. An antibacterial-loaded hydrogel coating may represent a possible option to protect orthopaedic implants from bacterial colonization, provided that further studies will confirm its efficacy in vivo, as recently reported [22], and in clinical trials.
